# Anaesthesia of three young grey seals (*Halichoerus grypus*) for fracture repair

**DOI:** 10.1186/2046-0481-64-3

**Published:** 2011-03-31

**Authors:** Vilhelmiina Huuskonen, Lynne Hughes, Rachel Bennett

**Affiliations:** 1 UCD Veterinary Hospital, School of Agriculture, Food Science and Veterinary Medicine, University College Dublin, Belfield, Dublin 4, Ireland

## Abstract

Three young grey seals (Halichoerus grypus) were presented separately for fracture repair to the veterinary teaching hospital of University College Dublin. The seals were premedicated with a combination of pethidine, midazolam and atropine; anaesthesia was induced with propofol via the front flipper vein and maintained with sevoflurane or isoflurane in oxygen. One of the seals did not breathe spontaneously after anaesthesia; a cardiac arrest, resulting in death, occurred after several hours of mechanical ventilation. Post-mortem examination revealed a severe lungworm infestation and parasitic pneumonia in this animal. The two other seals recovered uneventfully from anaesthesia.

## Background

*Pinnipeds *are a group of marine mammals divided into three groups: *Odobenids *(walruses), *Otariids *(eared seals, including sea lions and fur seals) and *Phocids *(earless or true seals including the grey seal). *Pinniped *anaesthesia can be challenging due to the unique adaptations of these animals to underwater life, i.e. cardiovascular, respiratory and thermoregulatory adaptations [1-5]. In addition, seals are wild animals and therefore are difficult to handle, which makes administering safe and successful anaesthesia difficult. The Irish Seal Sanctuary rescues and rehabilitates injured seals found on the Irish coast; they are often brought to UCD Veterinary Hospital for diagnostic work and surgery. This case series describes the anaesthetic management of three seals presented with fractures.

## Methods

### Description of the cases

Details of the seals, anaesthetic drugs and monitoring are included in Table 1.

**Table 1:** Details of the seals, anaesthetic protocol and monitoring

	Case 1	Case 2	Case 3
Age, sex, wt	6 weeks, female, 12 kg	8 weeks, male, 16 kg	2 weeks, female, 20 kg

Presenting complaint	Open mandibular fracture >1 week duration	5^th ^metatarsal bone fracture	Left tibia and fibula fracture > 4 days

Premedication	Pethidine^1^ (2 mg.kg^-1^), midazolam^2 ^(0.2-0.3 mg.kg^-1^), atropine^3 ^(0.02-0.04 mg.kg^-1^) IM		

Induction	Propofol^4 ^(1.25-2 mg.kg^-1^) IV 30-40 minutes after premedication		

Endotracheal tube	6.5 mm cuffed	7 mm cuffed	8 mm cuffed

Maintenance	Sevoflurane^5 ^in 100% O_2_, IPPV	Sevoflurane in 100% O_2_, IPPV	Isoflurane^6 ^in 100% O_2_, IPPV

End tidal anaesthetic agent	ET sevo 3-3.3%	ET sevo 1.7-2.7%	ET iso 0.5-1.4%

ETCO_2_	6.0-7.4 kPa	4.6-6.0 kPa	4.6-6.0 kPa

Fluid therapy	CSL + 2.5% glucose (10 ml.kg^-1^h^-1^)	CSL (10 ml.kg^-1^h^-1^) Bolus of 2.5% glucose (5 ml.kg^-1^)	CSL (10 ml.kg^-1^h^-1^) CSL + 2.5% glucose (10 ml.kg^-1^h^-1^) Hetastarch (1 ml.kg^-1^h^-1^) Hetastarch boluses (total 12 ml.kg^-1^)

Analgesia	Bupivacaine^7 ^(1 mg.kg^-1^) bilateral mandibular nerve block Meloxicam^9 ^(0.1 mg.kg^-1^) IV	Meloxicam (0.1 mg.kg^-1^) IV Lidocaine^11 ^(3.75 mg.kg^-1^) splash block	Butorphanol^10 ^(0.1 mg.kg^-1^) IV 90 minutes after premedication

Haemoglobin saturation (SpO_2_)	96% during surgery; 78% at the end of surgery	97-99%	97-99%

Heart rate	85-125 bpm	100-130 bpm	100-130 bpm

Body temperature	33.9-37°C	35.2-35.3°C	34.4-36.2°C

Blood glucose (minutes post induction)	7.6 mmol.l^-1 ^(10) 9.3 mmol.l^-1 ^(55) 10.3 mmol.l^-1 ^(100) 5.7 mmol.l^-1 ^(145)	6.6 mmol.l^-1 ^(20) 5.8 mmol.l^-1 ^(50) 5.1 mmol.l^-1 ^(80) 3.1 mmol.l^-1 ^(110)	6.1 mmol.l^-1 ^(15) 3.2 mmol.l^-1 ^(60) 3.1 mmol.l^-1 ^(105)

Duration of surgery	60 minutes	75 minutes	80 minutes

Duration of anaesthesia	90 minutes	125 minutes	150 minutes

Return to spontaneous breathing (minutes after end of anaesthesia)	Died	25 minutes	25 minutes

The seals were manually restrained and pre-medicated with pethidine¹ (2 mg.kg^-1^), midazolam^2 ^(0.2-0.3 mg.kg^-1^) and atropine^3 ^(0.02-0.04 mg.kg^-1^), intramuscularly (IM) into the post-scapular (case 1) or lumbar muscles (cases 2 and 3). When sedated, a 20 gauge intravenous (IV) catheter was placed in the palmar aspect of the front flipper. Anaesthesia was induced with an IV bolus of propofol^4^, and the airway was intubated with a cuffed endotracheal tube and the cuff inflated. Intubation was easy in all cases. Due to profuse salivation, case 1 received an additional dose of atropine (0.02 mg.kg^-1^) at the time of intubation. The endotracheal tubes were then connected to a circle breathing system and anaesthesia was maintained with sevoflurane^5 ^(cases 1 and 2) or isoflurane^6 ^(case 3) in 100% oxygen. Intermittent positive pressure ventilation (IPPV) was initiated immediately and continued throughout anaesthesia. Intravenous fluids (compound sodium lactate, CSL), supplemented with glucose as needed, were given throughout anaesthesia. In addition to CSL, a synthetic colloid (hetastarch) was administered to case 3. Parameters monitored during anaesthesia included end-tidal carbon dioxide (ETCO_2_) and anaesthetic agent concentrations, heart rate and rhythm, and respiratory rate; all recorded every five minutes. Oesophageal temperature was measured every 15 minutes and blood glucose concentration every 30-45 minutes. Haemoglobin saturation was recorded intermittently in case 1 (due to the nature of the fracture and surgery) or continuously in cases 2 and 3 using pulse oximetry with the probe attached to the tongue. Depth of anaesthesia was monitored, subjectively, by checking jaw tone, palpebral reflexes, eye position and response to painful stimuli. In case 3, blood pressure was measured non-invasively using an oscillometric method; the blood pressure cuff was placed around one of the front flippers.

#### Case 1

A six week-old grey seal was presented for repair of an infected open mandibular fracture of one-week duration. The seal had been treated with antibiotics and non-steroidal anti-inflammatory drugs at the Seal Sanctuary prior to referral. At presentation the seal was dehydrated with a purulent nasal discharge and a history of cough. Pre-anaesthetic physical examination was limited as the seal was aggressive. On auscultation lung sounds were harsh and crepitant. No radiographs (thoracic or mandibular) were taken prior to anaesthesia. Prior to surgery bilateral mandibular nerve block (bupivacaine^7^, 1 mg.kg^-1^) was carried out using the technique previously described in dogs [6]. At the end of surgery mechanical ventilation was continued pending return of spontaneous ventilation and/or consciousness. ETCO_2 _levels were allowed to increase, reaching a maximum value of 15.5 kPa, in an attempt to stimulate spontaneous ventilation. However, this manoeuvre proved unsuccessful and mechanical ventilation was subsequently resumed. The seal received two doses of doxapram^8 ^(0.5 mg.kg^-1^) IV 15 minutes apart, however, without success.

Three hours after the end of surgery the seal's heart rate increased to 120-135 beats per minute (bpm). Two hours later the heart rate decreased dramatically to 45 bpm and ETCO_2 _decreased to subnormal values until there was no carbon dioxide in the exhaled air. Soon after the drop in ETCO_2 _asystole was observed on the ECG. Resuscitation was not attempted due to the poor condition of the seal, and poor prognosis. At post mortem examination the seal was found to have a severe lung worm infestation and parasitic pneumonia.

#### Case 2

An eight-week old male grey seal appeared bright and alert on presentation. The seal had an infected bite wound on the left tail flipper that had been treated at the Seal Sanctuary with clindamycin and meloxicam. Radiographs of the tail revealed a fracture of the 5^th ^metatarsal bone. Thoracic radiographs were not taken. On auscultation, lung and heart sounds were normal.

#### Case 3

A two-week old female grey seal was referred for surgical repair of an infected left tibial and fibular fracture. The seal had been found four days earlier, and had been treated with amoxicillin-clavulanic acid and meloxicam. On presentation the seal was bright, alert and quite aggressive. On auscultation, lung sounds were considered harsh on the left side, and normal on the right side. The heart sounds were normal on auscultation. Ten minutes after administration of premedication thoracic and tail radiographs were taken. Radiographs revealed a mild interstitial pattern in left cranial lung lobe; otherwise the lungs appeared normal.

## Discussion

Seals are wild animals and they present a danger to their handlers. As all of these seals were rescue animals and fairly aggressive, it was difficult to perform a complete pre-operative assessment and therefore pre-existing clinical problems may not have been identified. Moreover, although lung auscultation can be useful in providing information about the respiratory system, interpretation is difficult due to the fact that seal pups have very irregular respiratory patterns with periods of apnoea alternating with rapid breathing [7]. In case 1, a severe lungworm infestation was noted at post-mortem examination, which was a significant contributing factor to the poor outcome. Harsh lung sounds and purulent nasal discharge were noted before sedation; thoracic radiography may have helped to identify the extent of thoracic abnormalities pre-operatively. The authors now recommend performing thoracic radiography in wild seals pre-anaesthesia, especially in animals with signs of respiratory disease at the time of presentation; particularly as bacterial and parasitic respiratory diseases are relatively common in wild seal pups [7].

Anaesthesia in pinnipeds presents specific problems due to their anatomical and physiologic adaptations to diving for extended periods, i.e. cardiovascular, respiratory and thermoregulatory adaptations [1-5]. Reported complications of pinniped anaesthesia include apnoea, bradycardia (heart rate < 45 bpm), prolonged recovery, hypo- and hyperthermia and death [1,4,8-11]. In all seals, we encountered hypercapnia, mild hypoglycaemia and prolonged recovery; in one case these complications contributed to the death of the seal.

Cardiopulmonary changes during diving, i.e. the so-called 'dive response', include apnoea and profound bradycardia, redistribution of blood flow whereby the circulating blood is mainly limited to the heart and brain, and venous constriction which helps maintain normal blood pressure [1,3,11-13]. These changes are controlled primarily by central mechanisms [3,5], but are also influenced by peripheral receptor activity. Sympathetic activity increases during diving, causing splenic contraction and vasoconstriction, while the pulmonary stretch receptor activity decreases causing vagally-mediated bradycardia [3,5]. It is believed that the dive response can be inappropriately activated during anaesthesia, thus contributing to some anaesthesia-related deaths of seals [1,14,15]. Consequently, several authors recommend adding atropine to anaesthetic protocols, to prevent or diminish the bradycardia [4,8,9,15]. However, atropine will not stop the sympathetic activation responsible for vasoconstriction and redistribution of blood flow [3], and some authors choose not to use it routinely without any apparent increase in adverse effects [7,11]. Bradycardia can also occur in anaesthetised animals as a result of cardiovascular depression caused by anaesthetic agents, or following hypoxaemia due to anaesthesia-induced respiratory depression. The heart rates of resting adult seals show considerable individual and species-specific variation, ranging between 45-140 bpm [1,2,11,12]. Paediatric seals have higher resting heart rates than adults. None of the seals in this study were bradycardic during the anaesthetic period, having heart rates ranging between 85-130 bpm with a normal rhythm.

In addition to cardiovascular adaptation to diving, pinnipeds exhibit specific physiological and anatomical adaptations. They have large respiratory reserve and efficiency: their tidal volume is approximately 47% of the total lung capacity, compared to only 16% in horses at rest [2]. Their ability to deep-dive necessitates physiological modifications enabling prolonged periods of apnoea, and this apnoeic response is often seen in association with handling [1] or during anaesthesia [1,3,8,11,14,16]. Unacceptably long periods of apnoea, whether due to an inappropriate dive response or anaesthetic agents, can lead to hypoxaemia and if left untreated, eventually to hypoxaemia-induced cardiac arrest. Therefore, it is important to constantly monitor the oxygenation of an anaesthetised patient. Pulse oximetry appeared to be a reliable method of monitoring oxygen saturation of the seals in this report.

Pinnipeds may have an increased tolerance to carbon dioxide, and high ETCO_2 _levels have been reported in some anaesthetised species [12,17]. However, Reed et al. [2] found that ETCO_2 _values in freely diving grey seals were 4.6 ± 0.38 kPa, i.e. in the same range as in terrestrial mammals. Consequently, it would be justified to apply IPPV in anaesthetised seals to maintain normocapnia. This is particularly important when inhalant anaesthetic agents are used due to their dose-dependent respiratory depressant effects [1].

The ETCO_2 _measurements obtained by sidestream capnography in isoflurane-anaesthetised harp seals were reported to approximate to the partial pressure of carbon dioxide in the arterial blood (PaCO_2_) [17]. This result is of practical importance, since capnography is relatively easy to perform in anaesthetised and intubated seals, whereas arterial blood sampling for blood gas analysis is difficult to perform due to the lack of readily accessible peripheral arteries. For the same reason routine blood pressure monitoring of anaesthetised seals (both invasive and non-invasive) is challenging [18]. The authors failed to obtain readings for blood pressure in the first two cases despite several attempts to apply a Doppler flow detector or an oscillometric device. In case 3, blood pressure was measured non-invasively using an oscillometric method; the blood pressure cuff was placed around one of the front flippers. Mean blood pressure was approximately 50 mmHg throughout surgery. Three hetastarch boluses (to a total of 12 ml.kg^-1^) were given, however, the seal was hypotensive (mean blood pressure < 60 mmHg) until the end of anaesthesia when it normalised. However, these low oscillometric blood pressure readings might not have been reliable.

Anatomical adaptations of pinnipeds include a fleshy pharyngeal region, which may lead to respiratory obstruction when muscular tone is reduced by sedation [3,4,8,11,14,15]. After premedication administration, all seals were left in a dim and quiet room, which was closely monitored from outside until they appeared sedated enough for intravenous catheter placement. In addition to the fleshy pharynx, pinnipeds have incomplete tracheal rings to allow the trachea to collapse during diving, and pre-thoracic tracheal bifurcation [3,5,8-11,15]. These structural differences make the risk of endobronchial intubation high and sedation of pinnipeds challenging. Pinnipeds have also a more flexible thoracic wall than terrestrial mammals [3,8,9], and an ability to collapse the air-exchanging portions of their lungs during diving to prevent gas exchange and possibly nitrogen narcosis [19], thus making manual or mechanical ventilation under anaesthesia potentially more difficult [8]. However, no difficulties were encountered during the ventilating of these seals when peak airway pressures of 10-20 cmH_2_O were used.

Normal body temperature for phocid seals was reported to range between 36.0-37.2°C [7]. Although anaesthesia-induced hypothermia is a recognised problem in seals [1], hyperthermia is an even more common anaesthetic complication in pinnipeds [3,8,10,20]. Hyperthermia has been reported in anaesthetics lasting for an hour and a half or longer [9,10], and can possibly be due to the anaesthesia-induced inability of the seals to cool themselves down [3]. Therefore, warming a moderately hypothermic animal (temperature 34.0-35.9°C) is not indicated if a long-lasting anaesthesia is anticipated [9]. Hyperthermia can occur also during the post-anaesthetic period, and seals recovering from sedation or anaesthesia should be kept wet and in as cool an environment as possible [3,10]. More severe anaesthesia-induced hypothermia (body temperature < 34°C) should however be treated with active warming. None of the seals in this report became hyperthermic or severely hypothermic requiring intervention.

The life span of a grey seal is approximately 25 years [21] and the pups suckle for an average of 17 days [22]. The three seals in this report could be defined as paediatric thus having an increased risk of hypoglycaemia during anaesthesia [23]. Serum glucose levels of free-ranging adult harp seals were reported to reach 6.5-10.5 mmol.l^-1^; glucose levels of paediatric harp seals were even higher (up to 12.9 ± 1.8 mmol.l^-1^). Although this relatively high glucose level can be partially explained by stress-induced hyperglycaemia, marine mammals do have higher serum glucose values compared to their terrestrial counterparts [24]. Based on this data the seals in this case report all became hypoglycaemic during anaesthesia; their blood glucose concentrations ranged between 3.1-10.3 mmol.l^-1^. Case 1 received 2.5% glucose added to its intravenous fluids from the beginning of the anaesthesia, but cases 2 and 3 received supplemental glucose only after their blood glucose levels had dropped to 3.1 mmol.l^-1 ^and 3.2 mmol.l^-1^, respectively. Arguably, both of these seals should have received glucose supplementation much earlier in the anaesthetic period.

Reported anaesthetic combinations in phocid seals include a variety of combination of anaesthetic drugs. Many authors recommend premedicating all seals with atropine prior to induction [3,4,10,15]. The use of alpha-2 agonists (xylazine and medetomidine) has been associated with undesirable side effects in seals, e.g. hyperthermia, bradycardia, decreased cardiac output, prolonged anaesthetic recovery and even mortalities [3,18], although some authors have used alpha-2 agonists successfully [7,16]. Pethidine-midazolam combination induces a deep sedation with relatively mild cardiovascular side effects in leopard (Hydrurga leptonyx), crabeater (Lobodon carcinophagus) and southern elephant seals (Mirounga leonina) [3,4,15,18]. Ketamine has been used in phocid seals with benzodiazepines, or more commonly in combination with alpha-2 agonists both via intramuscular and intravenous routes, for its minimal cardiopulmonary depressing effect [18]. Propofol produces short duration anaesthesia with rapid recovery in seal pups [18], but is uneconomical and impractical to use in large adults. The authors chose to premedicate these three young seals with pethidine-midazolam-atropine combination to have deep sedation to allow intravenous catheter placement ± radiography. Afterwards, propofol was administered for induction while an inhalant anaesthetic was used for maintenance of anaesthesia. Depth of anaesthesia was deemed optimal for a surgical procedure when jaw tone was relaxed, palpebral reflexes were negative and the eye was rotated ventrally, and the seal did not respond to painful stimuli. Two of the three seals received sevoflurane, and the third seal was anaesthetised with isoflurane. There was no appreciable difference in return to spontaneous breathing in the second or third seals: in both seals mechanical ventilation was continued until spontaneous ventilation resumed 25 minutes after turning off the vaporiser. The swallowing reflexes of the second and third seals returned within five and 12 minutes respectively; at this point the seals were extubated without any complications.

Post-surgical pain management is of major importance in all animal species. All three seals in this study had received meloxicam prior to referral. Postoperative analgesia was provided with additional doses of meloxicam^9 ^as appropriate. Meloxicam can be used in seals, at canine dosages, following trauma or surgery without adverse effects [7,18]. All seals were also premedicated with pethidine, an opioid analgesic and sedative, with a duration of action of 1.5-2 hours in domestic mammals. One paper describes analgesia lasting as long as four hours after an injection of pethidine in several species of pinnipeds [25]. In none of our cases did the duration of surgery exceed the duration of action of pethidine. A single dose of butorphanol 55 μg.kg^-1 ^IM was reported to induce detectable plasma levels for up to five hours post-injection in northern elephant seals (Mirounga angustirostris) [18]. Only case 3 received a 100 μg.kg^-1 ^dose of butorphanol^10 ^IV 90 minutes after pethidine administration. It might be useful to administer butorphanol to prolong analgesia in seals; however the authors can not base recommendations on one case only.

In conclusion, pethidine-midazolam-atropine followed by propofol and inhalational anaesthetics, produced reliable surgical anaesthesia for fracture repair in paediatric grey seals. Although one of the seals died, post mortem examination showed that it had a severe underlying condition making recovery from anaesthesia unlikely.

## Competing interests

The authors declare that they have no competing interests.

## Authors' contributions

VH carried out the anaesthetics, drafted the manuscript and carried out all revisions. LH helped to plan the anaesthetic protocols, supervised the anaesthetics and revised the manuscript. RB helped to draft and revise the manuscript. All authors read and approved the final manuscript.

## Appendix

### Footnotes

1. Pethidine hydrochloride: Pethidine, Antigen Pharmaceuticals Ltd, Roscrea, Co. Tipperary, Ireland

2. Midazolam: Hypnovel^®^, Roche Products Ltd, Welwyn Garden City, UK

3. Atropine sulphate: Atropine, Antigen Pharmaceuticals Ltd, Roscrea, Co. Tipperary, Ireland

4. Propofol: Rapinovet^®^, Schering-Plough Animal Health, Welwyn Garden City, UK

5. Sevoflurane: Sevorane^®^, Abbott Laboratories Ireland Ltd, Dublin, Ireland

6. Isoflurane: Vetflurane, Virbac Ltd, Suffolk, UK

7. Bupivacaine hydrochloride: Marcain^® ^Polyamp^® ^Steripack 0.5%, AstraZeneca UK Ltd, Luton, UK

8. Doxapram: Dopram^®^-V, Fort Dodge Animal Health Ltd, Southampton, UK

9. Meloxicam: Loxicom, Norbrook Laboratories Limited, Newry, Co. Down, UK

10. Butorphanol: Torbugesic^®^, Fort Dodge Animal Health Ltd, Southampton, UK

11. Lidocaine hydrochloride: Lidocaine hydrochloride 2%, B. Braun Medical Ltd, Dublin, Ireland
